# The Nomos of the University: Introducing the Professor’s Privilege in 1940s Sweden

**DOI:** 10.1007/s11024-018-9348-2

**Published:** 2018-02-19

**Authors:** Ingemar Pettersson

**Affiliations:** Department of Economic History, Science and Technology Studies Center, Uppsala University, Box 513, 751 20 Uppsala, Sweden

**Keywords:** Professor’s privilege, Sweden, Science policy history, University patents, Academic entrepreneurship

## Abstract

The paper examines the introduction of the so-called professor’s privilege in Sweden in the 1940s and shows how this legal principle for university patents emerged out of reforms of techno-science and the patent law around World War II. These political processes prompted questions concerning the nature and functions of university research: How is academic science different than other forms of knowledge production? What are the contributions of universities for economy and welfare? Who is the rightful owner of scientific findings? Is academic science “work”? By following the introduction of the professor’s privilege, the paper shows how spokespersons for the academic profession addressed such questions and contributed to a new definition of university science through boundary-setting, normative descriptions, and by producing symbolic relationships between science and the economy. The totality of those positions is here referred to as a “nomos” – that is: a generic and durable set of seemingly axiomatic claims about universities. This Swedish nomos, as it took shape in the 1940s, amalgamated classical notions of academic science as exceptional and autonomous with emerging ideas of inventiveness and close connections between academics and business. Crucially, though, the academic-industrial relations embedded in this nomos were private and individual, thus in sharp conflict with the ideas of entrepreneurial universities evolving globally by the end of the 20th century.

## Nomos

In 1949, a new principle was introduced in the Swedish Patent Act: “*Teachers at universities, colleges or other establishments that belong to the system of education should not be treated as employees in the scope of this law*” (SFS 1949:345: §1). In practice, the formulation meant that academic scholars were not subject to a new patent law which gave employers the intellectual property rights to inventions made by employees. Or in other words: a patent generated by academic research belonged to the scientist, not to the university. Today – the legislation is still in place – this exception for university scholars is known as the “professor’s privilege”^1^ and has emerged as a controversy in debates over academic science in Sweden over the last decades (see Petrusson 2007; Benner 2008: 53; Färnstrand-Damsgaard and Thursby 2013), surely an indication of how important questions surrounding the nature and values of academic science have become in the nascent “knowledge society”. This paper, however, is not about those questions in today’s science policy discourses. Its purpose, instead, is to examine the historical processes leading to the introduction of the professor’s privilege and to show how the legislation reflected a shift in the political and economic role of university science in Sweden around the 1940s.

The discussion will revolve around three themes, each covering crucial elements of the professor’s privilege. The *first* theme deals with the normative content concerning science and commercial activities that was materialized through the legislation. *Secondly*, the paper examines the “symbolic work” – definitions, assumed relations, and demarcations – underpinning the professors privilege. A *third* main theme is about the power relations that shaped and determined the new legislation. As an overall hypothesis I argue that these three components formed a discursive framework – an assemblage of norms, symbolic relationships and hierarchies here identified as a “nomos”. Although this primarily is a historical study, the notion of a university nomos is, as indicated by the final section, a fruitful concept for understanding how the politics of academic science were transformed in the post-war era and why the professor’s privilege has become a matter of controversy.

Power is an apt point of departure in the discussion over this nomos. Previous publications and investigations (e.g. Sandgren 1981; SOU 1996:70: 92; SOU 2005:95: 114; Brundenius et al. 2011; Lidhard and Petrusson 2012) draw the same conclusion: the professor’s privilege is closely associated with the notion of “academic freedom”. This is a valid interpretation but says little of the kind of freedom implied. I interpret the processes leading to the professor’s privilege as part of a professional strategy to gain jurisdiction (see Abbott 1988: 59–85) over what we might call the integrity/productivity intersection. The term is inspired by Guston’s (2000) concept of boundary organizations, but refers here specifically to the transaction zone between science and business where commercialization occurs through exchanges between academic integrity and economic productivity. By focusing on the jurisdictional power over these intersections, this study shows that the introduction of the professor’s privilege was not a simple safeguarding procedure aimed against commercialization. Rather, the core rationale of the legislation was about gaining control over the linkages between academic science and industrial application in a time when the system for science and technology in Sweden was changing rapidly.

Crucial for this transformation was that patenting of academic science for the first time surfaced as a political issue in Sweden, a process that merged two strongly contrasting modes of producing knowledge. A patent system, on the one hand, provides the right for an individual or a corporate personhood to have monopoly on an invention for a certain period of time, an institutional structure that in theory gives incentives for inventors or firms to make long-term investments in inventions (see e.g. Arrow 1962: 617). Academic science, on the other hand, seems to have an ethos that in many respects clashes with commercial principles implicit in patenting. Patent policies on science could, potentially, thus easily cause unease among scholars that see them as infringing on their professional ethos (see e.g. Bud 2008). An obvious reference when discussing any such ethos of science is Robert K. Merton’s (e.g. 1942/1973) CUDOS norms codifying science as a common resource – universal and open rather than local or private, free from personal or economic interest, an institution producing path-breaking and original knowledge rather than applications and commercial goods. This paper highlights another normative configuration that combined economic interests with academic autonomy. That is: “commercialization”, an anachronistic expression here I should emphasize, was in fact an important part of the reasoning behind the professor’s privilege. “Academic entrepreneurship”, to put forth another anachronism, actually played a key role in the introduction of the professor’s privilege.

To some extent, the process described in this paper fits well in the mainstream conception of the formation of science policy in World War II which laid the foundations for the organization of research in the post war era: distinct boundaries between “basic” research and other forms of knowledge production (e.g. Pielke 2012); notions of science as a “public good” (e.g. Guston and Keniston 1994: 6–8); the idea of science as the ultimate motor of progress (e.g. Forman 2007); the university as an elite institution becoming “universal” (Trow 2010) and “entrepreneurial” (Etzkowitz 2002) by the end of the century. But the configuration of virtues and arguments behind the professor’s privilege indicates also a more intricate story of an emerging hybrid role of the university scientist, partly integrated in older discursive frameworks – disinterested, pure and theoretical – and partly in ideals following the expansion of science and technology policies – commercial, innovative and entrepreneurial. An implication of the study, hence, is that it pinpoints a formative event related to the kind of process Aant Elzinga (1985, 1997) refers to as “epistemic drift” – how external criteria for political, social and economic relevance gradually have influenced the internal normative structures of science. This indicates that the moral framing of the academic scientists was a rather complex construction. As Steven Shapin shows in *The scientific life* (2008), the late modern scientist was not necessarily driven by pure academic virtues; by scraping off the surface of the claims for academic freedom inherent in the professor’s privilege a different image of academic science in the 20th century shines through.

One could discuss this heterogeneous normative content behind the professor’s privilege as a “moral economy”, a concept stemming from E.P. Thompson’s 1971 study of upheavals among peasants in 18th century England and their moral principles for fair price-setting. From the 1990s on, the concept has been used by historians of science (e.g. Shapin 1994; Kohler 1994, 1999; Daston 1995) to highlight normative guidelines and sociocultural practices of scientific work in a certain milieu, or, in some cases (e.g. Kauppinen 2014), more specifically how they relate to activities within or outside academia. Although the notion of moral economies has generated many interesting results in studies of science, it lacks important explanatory affordances in terms of power and hierarchies. Thus, I believe the concept of nomos works as a more distinct alternative. Nomos is commonly associated with Carl Schmitt’s *The nomos of the earth* of 1950 in which the concept is defined as a “first partition and classification of space” (1950/2003: 67), and a typical example of what nomos may imply is the recognition of national sovereignty (see Jacques 2015). Schmitt, a scholar of law and politics, used nomos to characterize a fundamental political logic of the world prevailing in certain historical epochs – the East-West power balance of the Cold War to mention one example from his writings (1950/2003: 353). Currently, nomos is becoming a generic concept in social science and the humanities, defined, for instance, as a “fundamental law” and “a legitimate principle of division” (Bourdieu 2000: 96–97), or simply a “normative order” (Lemm and Vatter 2014: 5).

Schmitt illustrated the power of nomos by comparing it to geopolitical barriers: “nomos can be described as a wall, because, like a wall, it, too, is based on sacred orientations”  (1950/2003: 70). This idea can be smoothly relocated to the area of science and politics where boundary-setting plays a constant and fundamental role. To interpret how a nomos was formed I use Gieryn’s (1995, 1999) concept of boundary-work; in its most rudimentary form a description of constructions and reconstructions of symbolic boundaries between science and non-science. The professor’s privilege was clearly constructed through boundary settings and I will show how it was based on a set of defining notions each constituting an emerging nomos of academic science in Sweden – for instance, that university research was governed primarily by the urge for scientific knowledge expansion itself, that academic science was not “work” in the common sense, and that transactions between scholars and business were private affairs. Conceptions like these worked as a cohort of axiomatic claims underpinning the recognition of academic autonomy (see e.g. Albert and Kleinman 2011: 269). Obviously, there is a linguistic connection to the university here; the notion of being autonomous – that is: auto-*nomous* – carries a rationale of an institution with significant normative momentum.

## The Elliot Suggestion

The interwar period witnessed a challenging discourse around scientific property. A key event in this context was a set of policy guidelines developed internationally in the early 1920s by the League of Nation’s Committee of Intellectual Cooperation, a predecessor of UNESCO led by distinguished scientific intellectuals such as Marie Curie, Albert Einstein and Henri Bergson. The venture was propelled by a need to update legal routines and principles for ownership of scientific discoveries, an issue following an evolving assumption of a close relationship between science and economic development. Most important, at least for this paper, was a subcommittee on scientific property chaired by the Italian senator and lawyer Francesco Ruffini. The committee presented a report in 1923, arguing for the introduction of property rights of scientific findings – a radical suggestion that opposed a generally observed distinction between invention and discovery. The report gained mixed reactions from the member states of the League of Nations, and from the U.S., as the process continued in the 1920s and 1930s. The project was complicated partly by divergent legislative systems of the member states, but not least because the suggestion for scientific property challenged predominant conventions on the nature of science (Miller 2008). In Sweden, the reactions to the Ruffini report followed this pattern when it was assessed by legal expertise: the mixture economic ownership and science was controversial and challenged legal moral as well as legal presumptions. The patent legislation of 1884, which was in force in Sweden at the time, prescribed, for instance, a substantial difference between science and invention: “The activity of the scientist is not directly aimed on economic advantage […]. The inventor strives primarily for profit” (von Ehrenheim 1878: 43). Gösta Eberstein, distinguished lawyer in the field of intellectual property, commented on the Ruffini report and the debate it had generated in a paper of 1925. He acknowledged the importance of the topic, but questioned nevertheless the application of scientific discoveries to intellectual ownership: “they are too important and have too vast consequences to be subject of a property right like the one given to the inventor” (Eberstein 1925: 251).

The international movement lead by Ruffini and the repercussions it had globally in the interwar period signify an ongoing hybridization in the symbolic configuration of academic, scientific, economic and technical modes of knowledge production, a process propelled by industrialization and an intensification of political efforts in science and technology. In this change, the question of ownership emerged as an urgent yet complicated issue. World War II became a pivotal moment in this transformation as many industrialized countries reinforced their public infrastructures for science and technology significantly during the crisis. Crucial in the Swedish context was the establishment of facilities for military research such as the Institute for Military Physics (*Militärfysiska institutet*) formed in 1941 and incorporated in the Swedish National Defence Research Institute (*Försvarets forskningsanstalt*) in 1945, an organization that became a giant in the research landscape of Sweden in the post-war era (Agrell 1989: 100–111). Less known about these research facilities, but of importance here, is a controversy generated over a specific war-time patent legislation (SFS 1942: 550) introduced to protect national security during the war. Representatives of state authorities argued that inventions crucial for society, most notably military technology, should be controlled and developed by public establishments – a way to ensure that these inventions were developed efficiently in Sweden, without dispersing to unfriendly nations. (SOU 1944:27: 18–21). To give legal priority to the employer was a radical suggestion in the tradition of patent law – the question had previously been tackled by a number of public committees over the interwar period, which all failed to come up with any new guidelines. Instead, most instances had ended up supporting an older legal practice according to which the employee formally had the rights to inventions but that allowed for employers to circumscribe the right in the employment contract. Swedish industries commonly used standardized formulas stipulating that inventions associated with the work of the employee belonged to the employer while the inventor was guaranteed a fair compensation (SOU 1944:27: 11, 15–16; Neumeyer 1963: 4–5).

In parallel with these events, the issue of employees’ patents was brought up in reforms carried out by one the most important actor in the organization of research in Sweden historically, *The Committee for the Organization of Techno-Scientific Research*, commonly called the “Malm Committee” with reference to its chairman, conservative politician and engineer Gösta Malm (see Stevrin 1978: 82–93; Weinberger 1996: 31–99). The Malm Committee faced the issue of intellectual ownership when it worked out a proposal on how to organize semi-public industrial research institutes, establishments for techno-science combining economic rationales of industry with principles of public administration, thus challenging prevalent legislation for patents. As a solution, the committee suggested that this could be achieved with a split organization with one privately funded unit that conducted commissioned research and allowed for private clients to have primary rights to patents, whereas another unit carried out state supported “basic research” and had the right to control patents itself (Pettersson 2012: 107–124; Pettersson 2016). An ingenious solution in many respects, but these institutes were special cases and their organizational model could not easily be applied to the growing range of public research organizations in Sweden. So when Secretary of Commerce Herman Eriksson elaborated on the issue in 1942, he concluded that ownership of public research was such a pressing issue that it required further scrutiny (Prop. 1942: 282).

The task was delivered to Knut Elliot, lawyer and state secretary at the Ministry of Justice, who was appointed in June 1943 to run a public investigation on inventions made by employees at public authorities. He presented a report in 1944 – and it was here that the original idea of a Swedish professor’s privilege, often referred to as the “Elliot suggestion”, was first articulated. The aforementioned law allowing the state to own and control inventions of importance for Sweden’s national security had influence on Elliot, but he took the reasoning of that legislation even further by suggesting that all public employers should own the patent rights for inventions made by employees. This was a radical suggestion in the context of previous law, but there was an important difference between private and public employers Elliot argued: regulations giving private employers ownership to employees’ inventions could seriously threaten technological renewal since private employers commonly lacked sufficient resources for development. The state, on the other hand, according to Elliot’s reasoning, had abundant resources and the necessary means to develop promising technology to its full potential, and should thus have the right to own and exploit inventions generated within the sphere of public authorities (SOU 1944:27: 5, 43).

As we shall see, Elliot’s ideas on public authorities and patents were a bit too drastic and became no great success. Ironically, however, it was the array of exceptions he was forced to formulate in order to make his radical suggestion viable that eventually gave his report significant historical impact. Some notes on those exceptions: Elliot’s proposition for a new law applied only to employees with research or other forms of inventive work as part of their employment, and it made clear also that it only concerned inventions associated with the main task of the employment. Another important exception in Elliot’s suggestion was that the law should only cover public authorities, not state *supported* organizations – an exception imposed by representatives of the state supported branch research institutes which, according to the arguments, needed sufficient legal space to manage relations with industries without interference from state regulations (SOU 1944:27, 5, 22). The main exception, however, at least in the scope of this study, concerned universities and other public organizations for education. These, Elliot argued, should be excluded from the legislation he suggested. We are hence at the very core of this article – the professor’s privilege – and the question I will discuss in the remaining part of this section is how Elliot framed this part of his legislation. The following list presents, based on my interpretation, the pillars of Elliot’s reasoning for excluding academic research from the proposed law (SOU 1944:27, 45–46, 55–56):

1. *University science is carried out without predefined causes or directions except for the goal to expand the base of scientific knowledge*. This shows an understanding of university research impossible to combine with the legislation suggested by Elliot, which held that the employer had the rights to inventions associated with the main task of the employment. In other words: if Elliot’s new legislation was applied without an exemption for academic research, the consequences would be almost absurd since the law then would give the university as employer a limitless right to inventions.
2. *University science is indistinguishable from academic education*. This essentially Humboldtian – no references in that direction were given by Elliot, nota bene – principle supported the point above. Higher education was understood as a venture guided by the idea of infusing students with a spirit of science; it could and should not be reduced to a specific work task. The educational mission gave academic science an exceptional position and demarcated universities from other public organizations carrying out research, such as the recently established branch research institutes and other public research establishments.
3. *University science is not work*. An important implication of Elliot’s reasoning was that academic science was carried out in ways and for reasons making it intrinsically different from other professions and occupations. This point of Elliot’s reasoning deserves specific attention here: university scholars were exceptional. University research was not “work”, nor a “job”, it was a professional trait requiring special arrangements and a large share of autonomy in order to function properly.
4. *The possibility of patenting is important for individual inventiveness in academic research*. University research was perceived by Elliot as a personal conduct and he argued that patent rights, if given to the university as an organization, would have negative implications on scholars’ initiative and creativity. This part of Elliot’s proposal aligned with the notion of academic freedom an idea of patenting as a driver of invention. Hence, the professor’s privilege was not an idealistic gesture of scientific pureness and calling as the points above may suggest; it was essentially about the freedom to be inventive. This principle is closely associated with the next:
5. *University-industry relations are affairs between the individual scholar and the client*. Commissioned research was not particularly unusual but was seen as an activity taking place outside the realm of the academic organization; it was an affair between an individual scientist and a firm. This argument should be seen in light of the circumstance that academic scientists could have financial support from private industries, or even taking part in business. Such arrangements were common, not least before the 1940s when public support of industrial science still was comparatively low (Althin 1970: 181). The close industrial collaboration the chemist and Nobel Prize winner of 1926, The Svedberg, had in the interwar period is one distinct example of how academic science and markets interacted (Widmalm 2006: 165–168; Bragesjö et al. 2012: 77–78). This says something of importance for this paper; commercial dispositions were indeed incorporated in the nomos as long as it was an individual conduct. The nexus of integrity and productivity was a private and individual domain where public organizations should not interfere.
6. *The university as an organization lacks sufficient routines to exploit inventions*. To the fifth point should be added an understanding of the capabilities and rationales of contemporary academia in Sweden. This shows an idea of the academic organization as intrinsically non-commercial.

Elliot’s report is a unique historical document as it produced distinctive legal definitions of academic science. Whereas this part, Elliot’s arguments for the professor’s privilege, was received well, other parts of the report met hard criticism when assessed by public and private authorities. A general objection, articulated by the Patent and Registration Office (*Patent- och registreringsverket*), claimed it was unpractical to have a patent law demarcating public from private employment, and argued for more coherent legislation. Some authorities were critical against regulations per se. The Swedish Federation of Industry (*Svenska industriförbundet*) and The Swedish Steel Producers’ Association (*Jernkontoret*), both powerful representatives of the Swedish industrial elite, argued that there was no need for regulation, assumedly as it would constrain employers’ possibilities to close deals directly with employed personnel. The newly established Swedish Confederation of Professional Employees (*TCO*, *Tjänstemännens centralorganisation*), on the other hand, dismissed Elliot’s suggestion and accused it of undermining the already weak position employed inventors had whereas the Swedish Inventor’s Association (*Svenska uppfinnareföreningen*) warned that the legislation would make it difficult to attract top scientists to public research facilities. Furthermore, a series of state authorities questioned the very core of Elliot’s report, that public employers had any reason to earn ownership over inventions made by employees (SOU 1946:21: 29–30). All in all, Elliot’s proposal seems to have raised more questions than it managed to clear out. 30 out of 60 official responses rejected his proposal (Prop 1949:101: 14).

But to be clear: the main point of interest here – Elliot’s idea of university science as exceptional to other forms of work and knowledge production – did not receive any substantial criticism, an indication that the professor’s privilege reflected prevalent norms and definitions of academic science. Considering the historical significance of Elliot’s reasoning, however, questions of his inspirations obviously occur. Unfortunately, neither his report, nor the archive of the committee give any clues about the sources of Elliot’s ideas, and there are no references to foreign patent legislation or other possible influences. Who was Elliot? It is difficult to establish if he was a strong proponent of academic values or if he just reflected values in the air at the time. What shows about his career in terms of publications and affiliations suggests that he was a typical civil servant: state secretary at the Ministry of Agriculture in the 1930s and later president of the Royal Court in northern Sweden (Thyselius et al. 1969: 237; Fock 2012: 214–215). There is nothing otherwise in the documentations of his career suggesting any particular sentiment for academic values. My explanation for the question of where the inspiration came from is that Elliot’s proposal reflected powerful ideas and values he was in contact with. Elliot was, as far as my interpretation goes, what Latour (2005: 38–39) calls an intermediary, an actor translating meaning without actively producing it – a spokesperson (see Akrich et al. 2002) of the nomos. Elliot’s historical significance in this case, hence, is not about the production of ideas, but of the packaging and the translation of the academic nomos into the codified realm of law. In the next section, I will give a clearer answer to the question of what movement Elliot spoke for.

## Aligning Universities with Economic Progress

What mechanisms produced this nomos? First of all, I wish to emphasize again that the legal and moral configurations of patents in science was in a state of flux, leaving the field open for opposing sets of interpretations and organizational solutions. In 1942, for instance, the Malm Committee argued, in favour of the idea of public research establishments as patent holders, that “patenting could provide research in Sweden with foreign capital and give institutions extra encouragement” (SOU 1942:6: 145). This opinion, resembling in key respects today’s notion of “entrepreneurial” universities, proved to be controversial, however; in 1944, Alf Grabe – an important actor: teacher in metallography at The Royal Institute of Technology and chairman of the Swedish Inventor’s Association – warned that such principles could corrupt the openness of research at public facilities: “research institutions are not meant to be patent-gathering establishments” (Svenska uppfinnareföreningen 1944: 521). Furthermore, in 1946, Gösta Malm, now chairman of the board of the Swedish Wood Research Institute, took a new position by pleading to the Minister of Justice that the newly established branch research institutes should be given the same legal status as academic establishments in the upcoming law for inventions and patents (KA-J 4). His claim, which radically pushed these institutes toward the realm of the academic, was not approved but illustrates nevertheless the turbulence of the ongoing negotiations on the boundaries of the research system. Secondly – and this will be the main theme of this section – I suggest that we see the rhetoric of Elliot’s work as a reflection of an emerging new position of academic science, a sign of an increasingly successful campaign for making academic establishments brighter symbols of material progression in Sweden. This position was far from evident at the time. Initially, the reforms of the research system were conveyed by a technoscientistic ethos prioritizing hybrid modes of knowledge production and organizations rather than “pure” university science (Pettersson 2016). But eventually, this project caused reactions. As an example, in 1944, the Chancellor of the Swedish universities, Östen Undén, argued that the academic sector needed better support:

At a time when the government has started to pay attention to the exceptional importance of intensified technical research for the country’s economy, it must be considered insufficient use of resources to compel university science departments to cut-backs which hamper the education and research taking place there. An indispensable condition for the efforts to strengthen technical research and promote our country is undoubtedly that a high standard is maintained in basic scientific research at universities and colleges. This requires sufficient funding however. (KFRU 1)

Undén’s claims indicate how political measures directed toward industrial techno-science impelled a campaign by the academic profession and gave rise to a new nomos that emphasized universities as motors of progress in society. Expressions of this reaction is found particularly in the arguments for the Swedish Natural Science Research Council (NFR, *Naturvetenskapliga forskningsrådet*), established in 1946. The process toward the formation of NFR was launched by two private members’ bills to the *Riksdag* (the Swedish parliament) in 1944. Harald Nordenson – who had significant network resources as conservative politician, industrialist and scientist, member of the Malm Committee and one of Svedberg’s successful pupils (see Widmalm 2006) – addressed a bill to the first chamber of the Riksdag backed up by a second bill from a group of conservative politicians. The bills confirmed that current efforts to strengthen industrial techno-science were important and necessary, but emphasized that technical progress would eventually run dry without a stable base of independent scientific research (Motion FK 1944: 238; Motion AK 1944: 357). Gösta Bagge, Minister of Education at the time, reinforced these arguments in his instructions for the committee that was appointed to investigate the establishment of NFR later in 1944: “improved working conditions for natural science are necessary for our country to maintain its cultural and material position internationally” (SOU 1945:48: 11).

The 1940s, and the war in particular, are an important passage in the history of the organization of science in Sweden as this period witnessed a political affirmation of a clear symbolic relationship between science, technical renewal and national prosperity. This symbolism brings us to a well-known figure in the history of science and technology, the so-called linear model of innovation. In its most elementary form, this model has been defined as the belief of science as a distinguished mode of knowledge production and a primus motor of technical and economic progress – a celebration of the pure and theoretical with long historical roots in Western culture (see Hounshell 2004; Godin 2006). In a stronger (see Balconi et al. 2010: 5) version the model suggests a chain-like path stretching from “pure” or “basic” modes of research, over technical application, to economic exploitation and development. Formalized models of such relations became commonplace from the 1960 and onwards – often inspired by OECD guidelines for statistical standardization and international comparisons (see Godin 2006) – but had no particular influence in the contexts we are interested in here.

This calls for some conceptual clarifications. The notion of a linear *model* is a rather blunt instrument for analyzing science policy in this context; it alludes to an idea of an explicit political blueprint difficult to find in historical material covering the earlier half of the twentieth century. In fact, the term “linear model” was not articulated in science policy discourses before the late 1960s (see Edgerton 2004: 34). Although a belief in science as an essential resource for modernizing and rationalizing industrial production shows clearly in the Swedish material of the 1940s, this cherished position of science was certainly not produced or influenced by any “model” for science. The position of science was rather the product of a multifaceted process in which scientists, politicians and industrialists mutually constructed meaningful and stable relations between science and society. Thus, it seems more accurate to describe the campaigns and reforms of the 1940s as an act of symbolic work establishing a linear order between science and economy. This reconceptualization – from model to symbolic order – sheds light on one the theoretical elements of this paper; controlling the integrity/productivity intersection was about keeping commercialization and other transactions of knowledge hidden under a veil of privacy. The linear order, hence, implied a rather enigmatic hierarchical relation between science and development in society – clear enough to be recognized by all relevant actors, hazy enough to elude policy and management.

A distinctive feature of the linear order ascending in the 1940s was the key position in which *academic* science was placed. NFR was essentially a support unit for universities – it was actually suggested that the council should be named “The University Board for Mathematics and Science” (“*Universitetsnämnden för matematisk och naturvetenskaplig forskning*”) before the term NFR was chosen (KFDNF 2). This aligning of academic science with material development deserves some further comments. The establishing of NFR was part of a longer historical trajectory where the role of universities changed from a more classic Humboldtian mode to entering a more distinct linear order in which academic research was seen as a strong force of production (e.g. Martin 2003). This transformation toward a new nomos, although it took radical steps forward in the 1940s, was incremental in a longer historical perspective and emerged as a crucial issue for academic scholars as universities were more closely tied to the Swedish state apparatus in the course of the 19th century. Stronger state support was often met with caution by academics fearing it could endanger the autonomy of the universities, institutions which traditionally had been financed through internal resources. Of key significance for this transformation of the Swedish universities is how professional autonomy was bolstered by the idea of research as a central task for academic science. By emphasizing that the subtle and internally driven practice of research was an indistinguishable and necessary element of teaching, the profession of academics could convincingly, and successfully, justify their autonomy and exceptional position (Blomqvist 1992: 47–51, 101–106).

This “researchification” of academic science was reinforced after the turn of the century. Early 20th century decades were characterized by an academic profession in Sweden aiming for a more stable position and a key event in that context was the 1916 introduction of research as a formal task for universities besides education (Elzinga 1993; Widmalm 2001: 131). But, to be clear, the introduction of research as a formal task is not to be seen as a complete entering into a linear order. In the interwar period, universities were still commonly seen as contributing to cultural rather than economic expansion, and it was not until after World War II that academic research finally was seen as a strong driver of material progress (see Elzinga 1993). This marriage of academic and economic came with a cost, however; alignment in a linear order required demarcations and a close-but-not-too-close position to the realms of business and politics. Examples of such strategies are found in the formal responses to the suggestion for NFR formulated by the Swedish Association for Junior Scientists (*Sveriges yngre naturvetares förening*) and the Swedish Federation of University and College Teachers (*Sveriges universitets- och högskoleamanuensers förbund*) in 1945. These associations acknowledged the benefits of closer contact between university science and business in Sweden, but emphasized than “an intimate relation between academia and industry must not disturb the universities' mission to conduct unbiased basic research” (KFDNF 1: 7). Reconnecting to Gieryn (1995: 434), this illustrates how boundary setting has been a way for scientists to protect their profession in times of increasing political intervention but simultaneously reap the benefits of the reforms – an act of “keeping politics near but out”. As we shall see in the next section, this tension between academic freedom and economic relevance, and the importance of recognized boundaries, became a salient concern when the political reforms for a new patent law entered a second phase.

## Deconstructing/Reconstructing Boundaries

On 16 March of 1945, about a year after Elliot had presented his report, a new committee was formed to bring clearness to the question of patents and employment. Elliot’s report had failed to give guidelines for new legislation and the task of the new committee was to take on the private as well as the public sector to introduce a more comprehensive solution. The committee was led by Nils Löwbeer, director-general of the Swedish Patent and Registration Office, and it presented a report in February 1946. Löwbeer’s proposal aimed for, allegedly, rational measures and diverged from Elliot’s in some important respects, particularly in terms of legislative coverage. Elliot’s idea of having specific legislation for public employments was abandoned in favour of a law covering all employments, a principle that clearly corresponded with the earlier opposition against Elliot’s proposal. Löwbeer argued, crediting prior critique, that the law Elliot suggested would have given improper advantage to the state and also made public employments less attractive for researchers and engineers (SOU 1946:21: 48). Löwbeer’s solution was to take Elliot’s overall ideas about ownership and apply it to both private and public employers, who were awarded ownership to inventions made within the frame of the employees’ work task or in other ways related to the production of the firm (SOU 1946:21: 43).

The task Löwbeer took on in 1945 proved to be rather complicated. Not only for reasons directly concerning university science and the nomos we are tracing here, but also because of reforms on the labour market. One such issue was that the collective actors on both parts of the labour market were against legislation per se and in favour of central agreements between employers and employees. The parts had already engaged in negotiations which had resulted in a general agreement about employments and patents between the Swedish Employer’s Organization (*SAF*, *Svenska arbetsgivarföreningen*) and the Swedish Union of Clerical and Technical Employees in Industry (*SIF*, *Svenska industritjänstemannaförbundet*) (Prop 1949:101).^2^ The deal between SAF and SIF was closed in January of 1946 but did not stop the introduction of a law, as we know. One can wonder why, considering the fact that the parts on the labour market had managed to solve the issue without state regulations. A likely explanation though is that the government wished to complete the larger restructuring of the patent law which had been initiated in the 1930s. Another explanation is that the agreement of SAF and SIF did not cover the whole labour market. The Minister of Justice, Herman Zetterberg, who eventually implemented the law in 1949, argued, for instance, that legislation was important since there were no agreements covering the sector of small-scale manufacture and craftwork (Prop. 1949:101: 53).

Another thing complicating Löwbeer’s investigation was employments at universities and colleges. A suggestion in the 1946 report, clearly in line with the aim for rational solutions, was to simply dismiss Elliot’s exception for universities. Löwbeer’s committee found no convincing arguments to why academic research would be damaged or endangered by being treated legally as wage labour in other areas:

The committee see no reasons, as suggested in the Elliot proposal, to exclude certain state institutions from the law’s scope of application. The committee would especially like to emphasize that the restrictions of employees’ rights the law might bring do not appear to impede scientific research at our universities and colleges. (SOU 1946:21: 51)

An interesting act of legislative boundary-work shows here. Elliot’s suggestion was based on an assumption of universities as intrinsically different and an idea that academic science could be harmed if it was not sheltered by institutional frameworks such as the professor’s privilege. Hence, it was an example of what could be called a “constructive” mode of boundary-work. Löwbeer’s committee, on the other hand, carried out a type of “deconstructive” boundary-work by blurring the demarcations around academic science established by Elliot two years earlier. It would be unfair to accuse Löwbeer’s committee to be hostile against universities or lack any appreciation of science as an institution; more accurately we may see the case as an indication that the normative order, the nomos, of the university was in a state of transition and it was still unclear how academic science should be dealt with in political reforms. About Löwbeer’s argument, it seems as the suggestion was based on an idea that the legislation would not have any significant impact on academic science, and therefore it made more sense for the committee to have a general law rather than one hacked up with exceptions.

But this was a miscalculation and an underestimation of the power of the nomos of academic science. Löwbeer’s committee left its proposal in 1946 but it took an additional time of three years to reach a final conclusion. Löwbeer’s suggestion that universities should be subject to the new patent law met strong, but not fully consistent, opposition. Chalmers Technical College (*Chalmers tekniska högskola*), the Karolinska Institutet, the faculty of medicine at Lund University, The College of Gothenburg (*Göteborgs högskola*), the College of Forestry (*Skogshögskolan*), The College of Agriculture (*Lantbrukshögskolan*) and The Institute of Odontology (*Tandläkarinstitutet*) did not raise any formal objections against the proposal. Protests, however, came from the medical faculty at Uppsala University, the section for mathematics and science at Uppsala University, the faculty of mathematics and science at The College of Stockholm (*Stockholms högskola*), and The Royal Institute of Technology (*Kungliga tekniska högskolan*) in Stockholm (Prop. 1949:101: 65). One can speculate why these establishments took this position, and why others did not. Possibly it was because that there was a strong community of academic scientists in the area – in the 1940s about 70% of all permanently employed university scholars were affiliated in the Stockholm-Uppsala region (Dahllöf 1980: 38). A qualified guess, thus, is that the opposition against Löwbeer’s suggestion had a stronghold in networks of scientists in the area. Uppsala University, as we shall see, was an important node in the upcoming critique.

We have now reached the finale of this story and I will give some indicative examples of how these actors articulated their position. There were, taken altogether, three core aspects of the argumentation given by the opponents of Löwbeer’s suggestion, all reflecting the ethos of Elliot’s prior declaration of the nature of academic science.

*First*, the university as an organization was seen as intrinsically non-commercial. The faculty of medicine at Uppsala University made clear that universities should not be involved in economic activities:

The proposed law, which primarily concerns industrial firms or public agencies with interest in larger-scale manufacturing of patentable technology, is not suitable for regulating the conditions at university departments conducting scientific research and education, and these departments should have no interest concerning commercial utilization of inventions made there. An application of the [suggested] law on university departments would lead to a situation where universities involuntarily would become stakeholders in, or even possessors of, patents; a consequence that would be problematic in several aspects. (KA-J 1)

*Second*, the economic rationale of patents was assumed of having negative influence on the independence of science. This was stressed by the section for mathematics and science at Uppsala University which referred to Elliot’s exception for universities as a better alternative than Löwbeer’s boundaryless proposal:

State secretary Elliot on the other hand suggests in § 1 of his investigation, that the law should not apply to universities and colleges and other establishments belonging to the educational system. The reason for this exemption is recognized as very legitimate by the section. Research at universities and colleges is, in contrast to investigations at commercial enterprises, public agencies and similar enterprises, not limited to specific needs. It is the nature of research to be free and independent. The section is convinced that an inclusion of the research at universities and colleges under the proposed patent legislation would have seriously negative consequences for the freedom of research. (KA-J 2)

*Third*, the “public good” character of academic science was emphasized. Here illustrated by the response to Löwbeer’s proposal written by substitute University Chancellor Thore Engströmer and addressed to the Minister for Justice:

With the deliverance of the received statements, I would like to express my support for them and emphasize that the proposed legislation is not applicable to the scientific institutions of education. At these, a more universal societal interest is promoted, directed toward the fostering of scientific research without immediate practical exploitation in mind. (KA-J 3)

This boils down to a matter of autonomy. The response to Löwbeer’s proposal from the section for mathematics and science at Uppsala University illustrates clearly how the academic profession protected the boundaries of university science and reinforced the nomos: “Every measure, which could lead to less independence for a scholar to dispose over and publicize its findings, must be resolutely dismissed” (KA-J 2). These objections obviously had influence on the Minister for Justice Herman Zetterberg who supported the arguments in his governmental bill. He concluded, without any elaborate reasoning, that: “teachers at universities, colleges or other establishments that belong to the system of education should not be seen as employees in the scope of the law” (Prop 1949:101: 66–67). Zetterberg’s bill was accepted by the Riksdag on May 24, 1949, without any substantial adjustments, and the new law for employee’s inventions was introduced by the beginning of 1950 (Rskr. 1949:320; SFS 1949: 345).

## Epilogue and Conclusions

From the framing of this article one could perhaps get the impression that the professor’s privilege was as debated when it was introduced as it has been over the last decades in Sweden. This was not really the case. The process described here was not marked by intense political controversy and I have found no systematically articulated sets of opinions from powerful actors such as industrialists or trade labour unions that, at least in theory, should have had an interest in the issue. One should note, however, that the professor’s privilege had important impacts in circles of law and inspired other countries. Similar legislation was introduced in Denmark in 1955, Finland 1967, Norway 1970 and in West Germany in 1957. These countries were eligible for this type of legislation since they in a similar way to Sweden had specific legislation for employees’ inventions, something which, for instance, the U.S. and the U.K. never had (SOU 1964:49: 15–17; Neumeyer 1977: 58) – an important factor outside of the scientific domain explaining why some countries have established versions of the professor’s privilege, and some not. Furthermore, the concentration of the professor’s privilege in the Scandinavian countries is explained by attempts in the 1940s to form a patent union and the broader tradition of legal cooperation between these nations (Neumeyer 1963: 35).

If we follow the political treatment of the professor’s privilege in the post-war era up until the 1980s, it will show that it was seldom, if ever, criticized in public policy debates. On the contrary, the rationales of the professor’s privilege were usually strengthened when investigated politically. In 1964, for instance, a state committee dealing with the legislation argued that the exception should not only apply to employees with teaching positions but also to a wider category of “scientific personnel” (SOU 1964:49: 37). Moreover, in 1980 another committee suggested to expand the exemption to all employees at public institutions for education (SOU 1980:42: 14). These reinforcements were, however, in essential respects legally redundant since the general interpretation of the law, already when it was introduced in 1949, included non-teaching personnel such as laboratory assistants (see Dennemark 1950: 20). A conclusion from this is that the nomos underpinning the professor’s privilege remained more or less uncontested up until and through the 1980s.

From the 1990s, however, the professor’s privilege has become subject of debate as it, according to an emerging critique, is an obstacle for making universities entrepreneurial and commercially oriented. A key inspiration in these contexts was the *Bayh-Dole Act* launched in the U.S. in 1980, which gave universities the right to take patents on state funded research, a breach with the former legislation that awarded the federal government ownership of inventions, and a formal consolidation of a trend in which universities became increasingly interested in holding patents (see Mowery et al. 2004; Popp Berman 2008). The Bayh-Dole Act gained strong influence outside the U.S. and has inspired an abolition movement that picked up pace by the end of the 1990s: Denmark abandoned the professor’s privilege in 1999 (Schovsbo 2008), Germany in 2002 (Kilger and Bartenbach 2002), Norway in 2003 (Iversen et al. 2007: 398) and Finland in 2007 (Oesch 2009; Veugelers et al. 2009: 295–295). This movement illustrates a change in which the intersection between the integrity and productivity of science has attracted attention from forces outside the academic profession, an element in the process of increasing political management of university-industry relations Geuna and Muscio (2009) refer to as an institutionalization of university knowledge transfer.

Although Sweden has taken a different path in this development, the professor’s privilege has been under considerable political pressure. An early example of critique in Sweden came from the economist Lennart Ohlsson who argued that the legal inconsistency between university inventors and inventors in non-academic settings caused a “market failure in an advanced economy” (Ds 1992:109: 211). The issue, according to Ohlsson, was that the professor’s privilege gave university scientists weak incentives and poor institutional support to commercialize their research and thus contribute to the industrial development of Sweden. Therefore, he recommended a Bayh-Dole type of legislation (Ds 1992:109: 212; Ohlsson 1993: 55–59). As a result of growing concerns about the professor’s privilege following this reasoning, a state committee was appointed in 2004 to investigate the possibility of dismantling the legislation, and suggested a removal to “provide a stronger spur for inventors to have the results of their research further developed into commercialisable products” (SOU 2005:95: 22). In 2008, however, the government decided that the professor’s privilege should be kept more or less in the original form of 1949 (Prop. 2008/09: 50) but introduced a number of organizational routines for making scholars responsible for reporting patentable inventions. The 2008 decision to keep the professor’s privilege did, however, not push the issue off the political agenda and it remained a political dilemma in Sweden how to combine policies for innovation with the older legislation. A committee report of 2012 concluded, for instance, that the professor’s privilege in combination with active support of innovation gave individual university scientists an unfairly privileged position on the market that potentially was in violation with EU decrees on open competition (SOU 2012:41 69–70).

For contemporary and historical reasons, Sweden has become recognized as a typical representative of the professor’s privilege and an opposite pole of U.S. policy on academic patents (Färnstrand-Damsgaard and Thursby 2013). This paper can only provide tentative historical answers to why this happened but an explanation must somehow take into account the wider political contexts in which the professor’s privilege was shaped. Previous studies on the breakthrough of science policies in the 1930s and 1940s argue that the emerging science and technology policies were instrumental in the political construction of a “Swedish model” of cooperation between the labour movement and the elites of industry (Nybom 1993: 166). It is not far-fetched to see connections between a nomos resistant to external intrusion in the affairs of academic commercialization, and Sweden’s relatively trustful labour market policies allowing trade unions and the employer’s main organizations to maintain and manage their internal affairs without intrusion by the government. Moreover, previous accounts of post-war science politics have highlighted the close connections of scientists and politicians in the foreground of the Social Democrats, notably long-lasting Prime Minister Tage Erlander – in office from 1946 to 1969 – who had studied physics at Lund University and kept close contact with the academic world throughout his career (Sandström 2000; Edqvist 2003). The impact of such relations should not be underestimated. The physics professor Torsten Gustafson, for instance, a close friend of Erlander, was a distinguished promoter of science in society: “*Framtidens Sverige kommer att bli forskningens Sverige*” – “Future Sweden will be a nation of research” (Gustafson 1956) is one of his more indicative formulations. Such attitudes and their proximity to the political elite shed light on the type of forces and connections that yielded the professor’s privilege. The nomos, and the cohort of norms, notions, hierarchies and symbolic capital it was made of, emerged in a policy atmosphere scented by optimism and faith in scientists and their promises.

The emerging debate surrounding the professor’s privilege is an indicator of how this nomos of the 1940s has been put under significant pressure during the drift towards commercialization and entrepreneurship in contemporary science politics (see Gibbons et al. 1994; Slaughter and Leslie 1997; Mirowski and Sent 2002). Important though is that the debate around the professor’s privilege should not be understood as a clash simply between proponents of commercially oriented academic science and defenders of autonomous academic science. The confrontation, as the paper suggests, is rather about different opinions on how the mechanisms of the integrity/productivity intersection are to be controlled – and by whom; the scholar or the manager? This paper shows, in line with what other accounts of post-war science policy in Sweden suggest (e.g. Jacob and Orsenigo 2007: 70–71), that individuality was a key feature of the academic nomos that was formed in the science policy reforms of the 1940s and has persisted into our days. The professor’s privilege did by no means isolate the academic profession from the economic sphere; it was not an upheaval against an emerging “Mode 2” (Gibbons et al. 1994) type of science policy emphasizing utility and application. On the contrary, the professor’s privilege ensured, legally, that academics could combine their ethos of science with the role of the inventor. It was, in a manner of speaking, a legislative tool letting the academic profession have their cake and eat it too; the professor’s privilege allowed the academic scholars to place themselves and the university in a key position in society without too seriously compromising the ideal of academic freedom. As the study shows, the nomos behind the introduction of the professor’s privilege combined “entrepreneurial” with academic, even Humboldtian, ideals; it included commercialization – or “academic entrepreneurship” to use a buzzword of today – in its bundle of fundamental norms. But importantly, it was entrepreneurship subordinated to academic freedom. The professor’s privilege facilitated private, not politically or managerially organized, economic transactions.

## Footnotes

1 There are alternative terms in use. “Teacher’s exemption” and “teacher’s exception” correspond directly to “*lärarundantaget*”, which is the most common expression in Sweden today. I use “professor’s privilege” (in spite of the slightly pejorative connotation) in this paper since it is most common in the international science policy discourse.

2 It is worth noting that this was a very Swedish solution: the consensus between the two parts shows a previously unrecognized, yet interesting, facet of the “Saltsjöbaden spirit”, the general compromising modus operandi characterizing relations between the main organizations of employers and employees from the 1930s over the post-war period in Sweden (see e.g. Johansson 1989).
